# A Novel Method for the Determination of Vitamin D Metabolites Assessed at the Blood-Cerebrospinal Fluid Barrier

**DOI:** 10.3390/biom11091288

**Published:** 2021-08-29

**Authors:** Sieglinde Zelzer, Andreas Meinitzer, Markus Herrmann, Walter Goessler, Dietmar Enko

**Affiliations:** 1 Clinical Institute of Medical and Chemical Laboratory Diagnostics, Medical University of Graz, 8036 Graz, Austria; andreas.meinitzer@medunigraz.at (A.M.); enko.dietmar@gmx.at (D.E.); 2 Institute of Chemistry, University of Graz, 8010 Graz, Austria; walter.goessler@uni-graz.at; 3 Institute of Clinical Chemistry and Laboratory Medicine, General Hospital Hochsteiermark, 8700 Leoben, Austria

**Keywords:** vitamin D metabolites, cerebrospinal fluid, blood-cerebrospinal fluid barrier, liquid chromatography-tandem mass spectrometry, biomarkers

## Abstract

The brain’s supply with vitamin D is poorly understood. Therefore, the present study aimed to determine 25-hydroxy vitamin D3 (25(OH)D) and 24,25-dihydroxy vitamin D (24,25(OH)_2_D_3_) in serum and cerebrospinal fluid (CSF) from individuals with intact and disturbed brain-CSF-barrier (BCB) function. In 292 pairs of serum and CSF samples the vitamin D metabolites were measured with liquid chromatography coupled to tandem mass spectrometry (LC-MS/MS). CSF/serum ratios (Q_ALB_, Q_25(OH)D_, Q_24,25(OH)2D3_) were calculated. Median (IQR) serum concentrations of 25(OH)D and 24,25(OH)_2_D_3_ were 63.8 (43.4–83.9) nmol/L and 4.2 (2.2–6.2) nmol/L. The CSF concentrations of both metabolites accounted for 3.7 and 3.3% of the respective serum concentrations. Serum 25(OH)D correlated inversely with Q_25(OH)D_ and Q_24,25(OH)2D3_ implying a more efficient transport of both metabolites across the BCB when the serum concentration of 25(OH)D is low. In patients with BCB dysfunction, the CSF concentrations and the CSF/serum ratios of both vitamin D metabolites were higher than in individuals with intact BCB. The CSF concentrations of 25(OH)D and 24,25(OH)_2_D_3_ depend on BCB function and the respective serum concentrations of both metabolites. Higher vitamin D metabolite concentrations in CSF of patients with impaired BCB function may be due to passive diffusion across the BCB.

## 1. Introduction

Vitamin D, a well-established regulator of calcium and phosphate metabolism, also has important functions in the central nervous system (CNS) [1]. Existing evidence indicates that vitamin D has neurotrophic and neuroprotective properties, and is involved in brain development [2]. Vitamin D supplementation seems to improve oxidative stress, inflammation, and cholinergic function in the CNS [3,4]. The vitamin D receptor (VDR) and the 1α-hydroxlyase, an enzyme that converts inactive 25-hydroxyvitamin D (25(OH)D) into active 1,25-dihydroxy vitamin D (1,25(OH)_2_D), are widely expressed in the brain [2,5]. Through the action of intracerebral 1α-hydroxlyase, 1,25(OH)_2_D can be synthesized locally. Previous studies have shown that vitamin D deficiency is associated with neurologic and psychiatric diseases including multiple sclerosis [6], dementia [7] and depression [8]. However, if these associations reflect a pathomechanistic involvement of vitamin D in these conditions, this is still a matter of ongoing debate.

Already in 1984, Balabanova S. et al. demonstrated that 25(OH)D, 24,25-dihydroxyvitamin D (24,25(OH)_2_D_3_), and 1,25(OH)_2_D are present in human cerebrospinal fluid (CSF) and that CSF concentrations of these metabolites correlate with those in serum [9]. The blood-CSF barrier (BCB) prevents the uncontrolled passage of circulating blood components into the extracellular fluid of the central nervous system. While some molecules can cross this natural barrier by passive diffusion, others undergo active and selective transportation. Until today, the transport of vitamin D metabolites across the BCB has been insufficiently explored. So far, only very few studies have measured 25(OH)D simultaneously in CSF and serum with inconsistent results [4,10,11,12]. For example, Lee et al. reported higher 25(OH)D concentrations in CSF than in serum, (10) whereas others found exactly the opposite [4,11,12]. Previous studies are limited by the use of analytical methods that have not been validated in CSF [13,14] and detected only 25(OH)D. Furthermore, assay comparison studies have shown substantial variability between different 25(OH)D methods [15,16]. Moreover, existing studies did not investigate the influence of BCB integrity on intracerebral vitamin D metabolism. It is important to mention that BCB dysfunction can impact the exchange of almost all molecules between the blood and the extracellular fluid of the central nervous system.

Over the last decade, liquid chromatography-tandem mass spectrometry (LC-MS/MS) has been established as the preferred method for vitamin D metabolite analysis as it offers superior sensitivity and specificity [17,18]. Furthermore, it allows the simultaneous determination of multiple metabolites [17,19,20]. So far, the utility of this technique for the measurement of vitamin D metabolites in CSF has not been studied systematically. Furthermore, the impact of BCB function on vitamin D metabolite concentrations in the intracerebral compartment is not known yet.

Therefore, the present study aimed to analyze the relationship between serum and CSF concentrations of common vitamin D metabolites with state-of-the-art technology. Furthermore, the impact of BCB function on vitamin D metabolite concentrations in CSF was studied using the CSF/serum ratio of albumin (Q_ALB_), a reliable and widely accepted surrogate marker of the BCB integrity [21].

## 2. Material and Methods

### 2.1. Patient Samples

During 2019, pairs of serum and CSF samples were collected from 292 consecutive patients who underwent lumbar puncture at the General Hospital Hochsteiermark at Leoben (Austria) for diagnostic purposes. The mean age was 53 ± 20 years. There were 146 (50%) males and 146 (50%) females. The final neurological diagnoses were of inflammatory (24%), vascular (19%), degenerative (18%), infectious (8%), idiopathic (7%), metabolic (3%), and neoplastic (3%) nature. No neurological diagnosis was discovered in 18%. CSF was collected in 2 mL VACUETTE^®^ Z No Additive tubes (Greiner Bio-one International GmbH, Kremsmünster, Austria). For serum, 5 mL VACUETTE^®^ Z Serum Clot activator tubes (Greiner Bio-one International GmbH, Kremsmünster, Austria) were used. For routine analyses, samples were kept at 4 °C for maximum seven days and residual samples were deep frozen at −80°C until batched analysis.

BCB integrity was assessed by the albumin CSF/serum ratio (Q_ALB_) according to Reiber et al. [21,22]. In addition, the CSF/serum ratios for 25(OH)D (Q_25(OH)D_) and 24,25(OH)_2_D_3_ (Q_24,25(OH)2D3_) were calculated in all samples. Patients <18 years of age were excluded from the study. Informed consent was obtained from all patients. The study was approved by the local ethics committee [7] of the Medical University Graz (Graz, Austria; EC-number: 31-391 ex 18/19) and carried out in accordance with the standards of the declaration of Helsinki.

### 2.2. Measurement of 25(OH)D_3_, 25(OH)D_2_ and 24,25(OH)_2_D_3_

The vitamin D metabolites 25(OH)D_3_, 25(OH)D_2_ and 24,25(OH)_2_D_3_ were analyzed with an LC-MS/MS method that has been recently validated for serum [19]. For measurement of CSF samples, the same method was re-calibrated with CSF-based calibrators and the analytical performance in this matrix was evaluated according to the recommendations of the Food and Drug Administration [23]. Briefly, after sample preparation, including protein precipitation (potassium hydroxide) and liquid/liquid extraction (n-heptane:tert-methyl-butyl-ether, 1 + 1) followed by derivatization with 4-phenyl-1,2,4-triazoline-3,5-dione (PTAD), samples were separated on a Nexera UHPLC from SHIMADZU (Kyoto, Japan) using a Kinetex^®^ 5 µm F5 100Å LC column (150 × 4.6 mm, Phenomenex, Torrance, CA, USA) with gradient elution. A SCIEX QTRAP 6500 triple quadrupole instrument (Applied Biosystems, Framingham, MA, USA) was employed for detection. For measurement in serum and CSF 50 µL and 200 µL of sample were used. The method was calibrated with 6 calibrators that were prepared by spiking a 7% human albumin solution with six concentrations of standards from 25(OH)D_3_, 25(OH)D_2_ and 24,25(OH)_2_D_3_, together with the internal standards (ISTD) whereby d6-25(OH)D_3_ was used for 25(OH)D_3_, d3-25(OH)D_2_ for 25(OH)D_2_ and d6-24(R),25(OH)_2_D_3_ for 24,25(OH)_2_D_3_. For each calibrator the analyte/ISTD peak area ratio was plotted against the nominal concentration of each compound [19].

The CFS calibrators were prepared by spiking aliquots from a CSF pool with different amounts of 25(OH)D_3_, 25(OH)D_2_ and 24,25(OH)_2_D_3_ (Table 1). To account for the vitamin D metabolites that are naturally present in the CSF pool, a blank correction was performed by subtracting the signal of the native CSF pool from the signal obtained after spiking.

The total 25(OH)D concentration was calculated by adding the concentrations of 25(OH)D_3_ and 25(OH)D_2_. The concentrations of 25(OH)D and 24,25(OH)_2_D_3_ in serum and CSF were used for calculating the respective CSF/serum ratios (Q_25(OH)D_ and Q_24,25(OH)2D3_). These ratios are estimates of the vitamin D metabolite transport across the BCB.

The performance of our LC-MS/MS method in CSF was evaluated according to the recommendations of the Food and Drug Administration [23]. The following performance criteria were determined: limit of detection (LoD), limit of quantitation (LoQ), linearity, intra- and inter-assay precision, and recovery. LoD was established by measuring the lowest concentration that produced a signal at least three times higher than the average background noise. LoQ was defined as the lowest concentration that allowed quantification with a precision of <10% [24]. Both indices were determined by analyzing serial dilutions of the lowest calibrator. Each dilution was measured five times.

### 2.3. Measurement of Albumin and Estimation of BCB Function

Albumin was determined by nephelometry on an Atellica^®^ NEPH 630 analyzer (Siemens Healthineers, Erlangen, Germany). Intra- and inter-assay coefficients of variation (CVs) ranged between 2.7–3.1 and 1.7–3.5%. The CSF/serum ratio of albumin (Q_ALB_) was calculated as a surrogate marker of BCB function [25]. According to Reiber et al. [21,22], the upper limit of reference for Q_ALB_ increases with age: 5.0 × 10^−3^ for patients <15 years, 6.5 × 10^−3^ for patients <60 years, and 8.0 × 10^−3^ for patients ≥60 years. Values above these cut-offs represent a disturbed BCB function.

### 2.4. Statistical Analysis

First, the distributions of 25(OH)D, 24,25(OH)_2_D_3_ and albumin concentrations in serum and CSF were tested for normality using the Kolmogorov-Smirnov test. Descriptive statistics are presented as medians and interquartile ranges (Q1–Q3). Correlations were performed with the Spearman’s rho (*ρ*) test. Linear regression models were calculated to describe the associations between Reiber scheme and the vitamin D metabolite ratios. The exact Mann-Whitney U test and the Kruskal-Wallis test were used for group comparisons. The post hoc test between medians of subgroups was calculated with Bonferroni. A *p*-value < 0.05 was considered statistically significant. All statistical analyses were performed with Analyse-it^®^, version 4.92 (Analyse-it Software, Ltd., Leeds, UK) and SPSS 20.0 (SPSS Inc., Chicago, IL, USA).

## 3. Results

### 3.1. Descriptive Statistics

During the 12-month recruitment period, 292 patients with an average age of 53 ± 20 years, were included. Males and females were equally distributed. Descriptive statistics are shown in Table 2. Based on the Q_ALB_, 117 patients had a BCB dysfunction whereas in 175 patients the BCB was intact. Amongst the 117 patients with BCB dysfunction, a definitive cause was identified in 97 individuals. The conditions associated with BCB dysfunction belonged to the following groups: inflammatory diseases (*n* = 28), vascular diseases (*n* = 22), degenerative diseases (*n* = 21), infectious diseases (*n* = 13), neoplastic diseases (*n* = 6), metabolic diseases (*n* = 4), and idiopathic causes (*n* = 3). In 20/117 (17.1%) of the patients with BCB, no definitive cause could be identified.

The median serum concentrations (IQR) of 25(OH)D and 24,25(OH)_2_D_3_ were 63.8 (43.4–83.9) nmol/L and 4.2 (2.2–6.2) nmol/L (Table 2). The CSF concentrations of 25(OH)D and 24,25(OH)_2_D_3_ were 3.7 and 3.3% of the serum concentrations.

### 3.2. Performance of the LC-MS/MS Method for Vitamin D Metabolite Measurement in CSF

The LC-MS/MS method is capable of detecting 25(OH)D_3_, 25(OH)D_2_ and 24,25(OH)_2_D_3_ in CSF. The retention times of 25(OH)D_3_, 25(OH)D_2_ and 24,25(OH)_2_D_3_ were 8.3, 8.5, and 6.2 min. The retention time of a possible isobaric interference of 1,25(OH)D was at 6.8 min in a high concentration range (100–200 pg/mL). Due to the low endogenous concentration, no 1,25(OH)_2_D could be detected. Representative chromatograms of 25(OH)D_3_, 25(OH)D_2_ and 24,25(OH)_2_D_3_ are shown in the supplementary material Figure S1. Serial dilution experiments of spiked CSF samples showed linear ranges that covered the concentrations of both metabolites in patients with and without BCB dysfunction (Table 1). The coefficient of determination (*r*^2^) was ≥0.997 for 25(OH)D and 24,25(OH)_2_D_3_. Intra- and inter-assay imprecisions ranged between 1.9–8.7% and 1.5–9.6%, respectively. For all metabolites, LoD ranged from 0.03 to 0.35 nmol/L and LoQ ranged from 0.05 to 0.70 nmol/L, respectively. Recovery ranged between 91.5% and 92.9% for all metabolites.

### 3.3. Impact of Serum 25(OH)D on Vitamin D Metabolites in CSF

**Figure 1:**
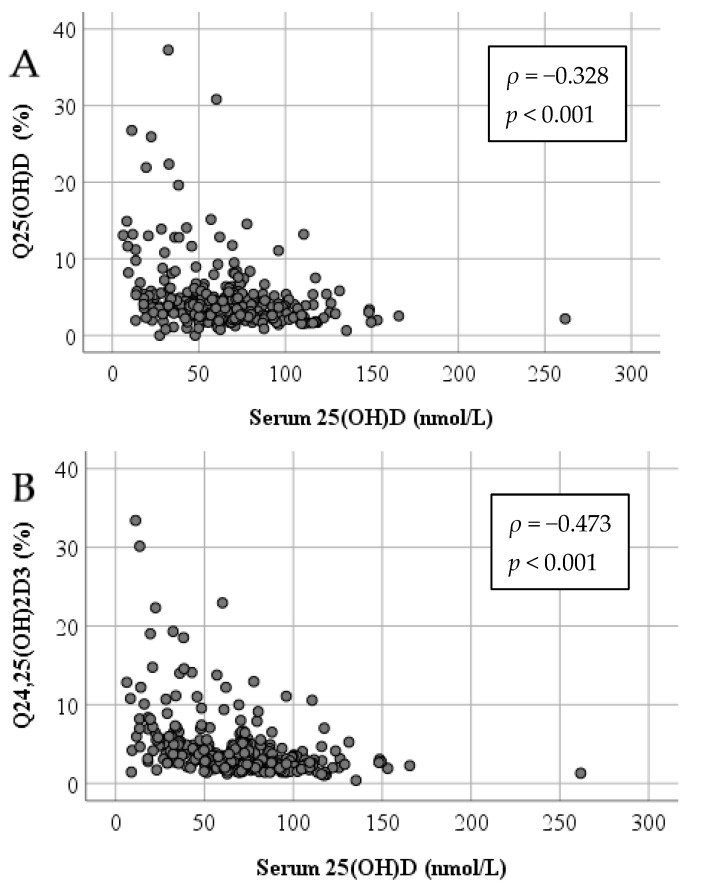
Correlations (Spearman’s *ρ*) between the serum concentration of 25(OH)D and the CSF/serum ratios of (**A**) 25(OH)D (Q_25(OH)D_) and (**B**) 24,25(OH)_2_D_3_ (Q_24,25(OH)2D3_) expressed as percent.

With decreasing serum 25(OH)D concentrations Q_25__(__OH)D_ (*ρ* = −0.328, *p* < 0.001) and Q_24,25(OH)2D3_ (*ρ* = −0.473, *p* < 0.001) increased significantly (Figure 1). In vitamin D deficient patients with serum 25(OH)D concentrations <30 nmol/L, Q_25__(__OH)D_ and Q_24,25(OH)2D3_ were approximately 55–75% higher than in vitamin D sufficient patients (Table 3). The inverse relationship between serum 25(OH)D and Q_25__(__OH)D_, and Q_24,25(OH)2D3_ was also present in individuals with BCB dysfunction. Of note, the serum 25(OH)D concentration was not significantly associated with the BCB function, as expressed by Q_ALB_.

### 3.4. BCB Function and the Relationship of Vitamin D Metabolites in Serum and CSF

**Figure 2:**
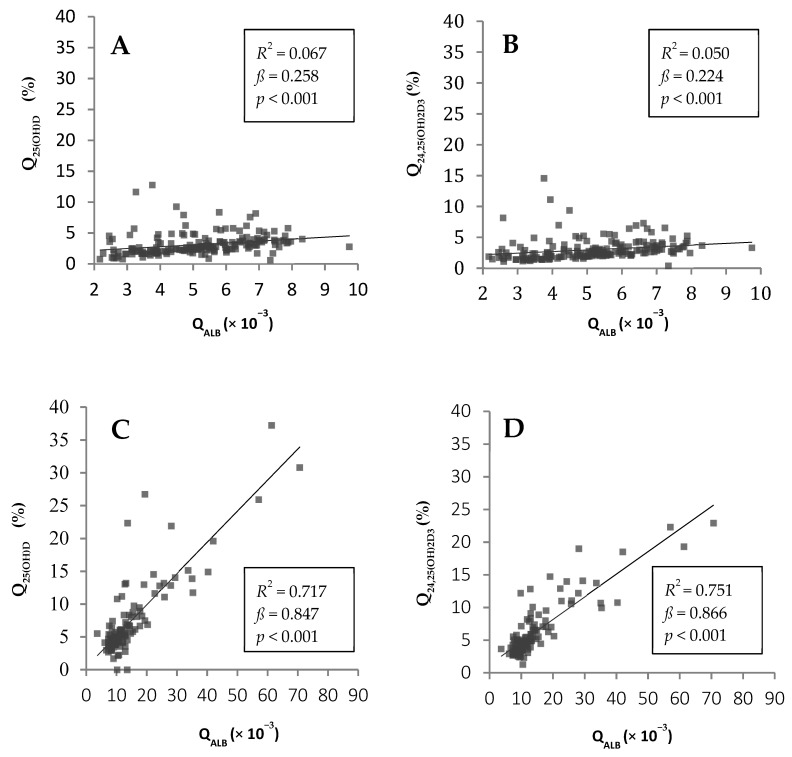
Univariate regression models between blood-CSF barrier (BCB) function (Q_ALB_, × 10^−3^) and the cerebrospinal fluid (CSF)/serum ratios of 25(OH)D (Q_25__(__OH)D_) and 24,25(OH)_2_D_3_ (Q_24,25(OH)2D3_) in patients without (*n* = 175) (**A**,**B**) and with (*n* = 117) (**C**,**D**) BCB dysfunction.

The median CSF concentrations of 25(OH)D and 24,25(OH)_2_D_3_ were significantly higher in patients with BCB dysfunction than in those without. Figure 2 illustrates that Q_25__(__OH)D_ and Q_24,25(OH)2D3_ increased with decreasing BCB function. In patients with BCB dysfunction, the associations between Q_25(OH)D_ and Q_ALB_ as well as Q_24,25(OH)2D3_ and Q_ALB_ were much stronger with markedly higher ß-values than in subjects with an intact BCB.

## 4. Discussion

The present study shows that the concentrations of 25(OH)D and 24,25(OH)_2_D_3_ are much lower in CSF than in serum. Furthermore, the CSF concentrations of 25(OH)D and 24,25(OH)_2_D_3_ are inversely related to serum 25(OH)D indicating an increased transfer of these metabolites across the BCB in vitamin D insufficient or deficient individuals. Dysfunction of the BCB leads to an increase of all metabolites in CSF. Furthermore, the tightly regulated passage of 25(OH)D and 24,25(OH)_2_D_3_ across the BCB is profoundly disturbed in patients with BCB dysfunction.

Our results are in contrast to a recent study by Lee et al. that measured 25(OH)D simultaneously in CSF and serum [10]. This study found higher 25(OH)D concentrations in CSF than in serum. However, in both matrices 25(OH)D was determined by a fully automated electrochemiluminescence binding assay from Roche Diagnostics that has not been validated in CSF. The LC-MS/MS method employed in the present study was validated for the use in CSF prior to the analysis of study samples. The results of this validation show good linearity, accuracy, precision, and recovery. Moreover, our method performs very well in the Vitamin D External Quality Assessment (DEQAS) program for the measurement of vitamin D metabolites in serum. Previous studies support a substantial variability of 25(OH)D measurements in CSF performed by either the electrochemiluminescence binding assay from Roche Diagnostics [4] or LC-MS/MS [11,12]. The present 25(OH)D results in CSF are well aligned with those from Holmøy T et al. which were also determined by LC-MS/MS. Moreover, the thorough validation in both matrices lends additional credibility to our LC-MS/MS method. Unfortunately, our LC-MS/MS method is not sensitive enough to measure 1,25(OH)_2_D, which circulates in picomolar concentrations in the blood and is found in even lower concentrations in CSF.

The present data support an active transport of 25(OH)D and 24,25(OH)_2_D_3_ across the BCB that depends on the serum concentration of 25(OH)D. Higher Q_25(OH)D_ and Q_24,25(OH)2D3_ values with decreasing serum 25(OH)D concentrations indicate that vitamin D deficient individuals upregulate the transport of these inactive metabolites across the BCB. These findings suggest a tightly regulated vitamin D metabolism in the CNS. In conjunction with numerous clinical studies that link vitamin D deficiency to neurological [7] and psychiatric disease [8], our results further support an important role of vitamin D for brain function and neuropsychiatric health. The concept of a functional role of vitamin D in the CNS is further strengthened by the expression of the 1α-hydroxylase in the cerebral cortex and the cerebellum [26,27]. Moreover, the vitamin D receptor (VDR), which is also present in human brain [5,28,29], mediates multiple vitamin D effects on cell proliferation, differentiation, and immune function [29,30].

The BCB is a complex structure [21] that can be crossed by blood compounds through passive diffusion (e.g., albumin, immunoglobulins) or active transportation (e.g., glucose, drugs) [31]. Until today, the transport of vitamin D metabolites across the BCB has not been studied systematically. However, recent functional experiments by Vernetti L et al. demonstrated the passage of vitamin D through the blood brain barrier [32]. In the present study (Figure 2), the slope of the lines of identity suggests that both vitamin D metabolite pass the BCB less efficiently than albumin. On the one hand, this could be explained by a protein-bound or lipoprotein-bound transport of vitamin D metabolites across the BCB where the resulting molecule-sizes are larger than that of albumin. On the other hand, in case that vitamin D metabolites cross the BCB in their free form, the very high affinity to VDBP could explain this observation. The present results demonstrate that dysfunction of the BCB strongly increases the passage of 25(OH)D and 24,25(OH)_2_D_3_ from blood into the intracerebral space. This is shown by substantially higher CSF/serum ratios for both metabolites. It can be speculated that in such patients, passive diffusion of vitamin D metabolites into the intracerebral space overwhelms the physiological transport of vitamin D across the BCB. Interestingly, an increased transport of 25(OH)D and 24,25(OH)_2_D_3_ into the brain is also visible in vitamin D deficient patients with BCB dysfunction. Previous studies, that determined vitamin D simultaneously in CSF and serum of patients with Alzheimer’s disease or multiple sclerosis, did not investigate the shuttling of vitamin D metabolites across the BCB [4,11,12]. Therefore, a comparison of our results with other studies is difficult.

Since the blood CSF barrier is a complex structure with manifold intercellular connections (e.g., tight junctions, gap junctions) combined with different CSF flow dynamic in the various anatomic structures and locations (e.g., ventricles, cisterns, lumbar and cortical region), the increase of protein and vitamin D concentration in CSF cannot simply be interpreted as a “leakage” or “barrier breakdown [21,33]. The Q_ALB_ is a representative tool to assess the permeability status in the context of these multifactorial influences at the blood-CSF barrier [21]. It must also be mentioned, that other derivates of hydroxyvitamin D or related compounds (e.g., lumisterol derivates) might be present in the samples [34,35].

Our study has some strengths and weaknesses that should be considered when interpreting the present results. The number of patients is substantially larger than in all previous studies, which ensures sufficient statistical power. Moreover, 40% of our patients had BCB dysfunction, which enabled us to investigate the role of this natural barrier in the vitamin D metabolism of the brain. 25(OH)D and 24,25(OH)_2_D_3_ were determined with a fully validated and rigorously controlled LC-MS/MS in both matrices ensuring validity of the results. Unfortunately, our method did not allow a reliable measurement of 1,25(OH)_2_D, which hampers a broader evaluation of the vitamin D metabolism in the brain. The use of a dedicated method for the measurement of 1,25(OH)_2_D was impeded by the small volume of CSF samples.

Some limitations of this study have to be mentioned. Information regarding pre-existing conditions, genetic variations, medication and body mass index were not collected. Furthermore, another weakness is the small samples size of only 117 patients with BCB. Finally, data on the 1,25(OH)_2_D metabolite are lacking.

In conclusion, with the LC-MS/MS method used in this study, 25(OH)D and 24,25(OH)_2_D_3_ can reliably be measured in plasma and CSF. The concentrations of 25(OH)D and 24,25(OH)_2_D_3_ are much lower in CSF than in serum. BCB function and the serum concentration of 25(OH)D are important determinants of the vitamin D metabolite concentrations in CSF. With decreasing 25(OH)D serum concentrations the transport across the BCB becomes more efficient. Higher vitamin D metabolite concentrations in the context of BCB dysfunction may be due to passive diffusion across the BCB. Future studies should further explore the regulation of vitamin D metabolism in the brain by measuring 1,25(OH)_2_D in plasma and CSF and relate the results to BCB function and the plasma 25(OH)D concentration.

## Figures and Tables

**Table 1:** Analytical performance of the vitamin D metabolites in CSF with LC-MS/MS method.

Vitamin D metabolites	25(OH)D_3_	25(OH)D_2_	24,25(OH)_2_D_3_
*m*/*z* ratio	558.4/298	570.2/298	574.2/298
Linear range (nmol/L)	7.8–250	1.5–48	1.5–48
Correlation coefficient (r^2^)	0.999	0.997	0.998
Intra-day precision (CV%)	2.2–5.9	1.9–8.4	3.4–8.7
Inter-day precision (CV%)	1.5–4.7	2.8–4.1	4.2–9.6
LoD (nmol/L)	0.35	0.1	0.025
LoQ (nmol/L)	0.7	0.7	0.05
Recovery (%)	92.1	92.9	91.5

*m*/*z*, mass to charge ratio; LoD, limit of detection; LoQ, limit of quantification; CV, coefficient of variation.

**Table 2:** Baseline characteristics of the study population.

Parameter		All Patients	Without BCB Dysfunction	With BCB Dysfunction	*p*-Values		
		(*n* = 292)	(*n* = 175)	(*n* = 117)	P1	P2	P3
Albumin	Serum (g/dL)	4.1 (3.7–4.4)	4.2 (3.9–4.5)	3.9 (3.6–4.3)	<0.001	<0.001	<0.001
	CSF (g/L)	0.27 (0.20–0.38)	0.21 (0.17–0.25)	0.40 (0.34–0.58)			
	Q_ALB_ (× 10^−3^)	6.8 (4.9–9.7)	5.2 (3.9–6.4)	10.8 (8.9–14.6)			
25(OH)D	Serum (nmol/L)	63.8 (43.4–83.9)	66.9 (46.6–87.7)	60.0 (38.2–76.1)	0.038	<0.001	<0.001
	CSF (nmol/L)	2.1 (1.5–3.5)	1.8 (1.3–2.5)	3.3 (2.2–4.8)			
	Q (%)	3.7 (2.4–5.4)	2.7 (2.1–3.7)	5.3 (4.1–8.1)			
24,25(OH)_2_D_3_	Serum (nmol/L)	4.2 (2.2–6.2)	4.4 (2.4–6.4)	3.6 (2.1–5.9)	0.102	<0.001	<0.001
	CSF (nmol/L)	0.12 (0.08–0.18)	0.10 (0.08–0.15)	0.18 (0.10–0.31)			
	Q (%)	3.3 (2.4–6.2)	2.6 (2.0–3.5)	4.9 (3.7–7.4)			

Comparison of serum concentrations (P1), CSF levels (P2), and CSF/serum quotients (P3) between individuals with and without blood-CSF barrier (BCB) dysfunction. Data are given in medians and interquartile ranges (Q1–Q3). The exact Mann-Whitney U test was used for subgroup comparisons. A *p*-value < 0.05 was considered statistically significant. CSF, cerebrospinal fluid; Q, CSF/serum quotient.

**Table 3:** CSF concentrations of vitamin D metabolites according to tertiles of 25(OH)D serum levels.

Parameter	All Patients, *n* = 292			Without CSF-Blood Barrier Dysfunction, *n* = 175				With CSF-Blood Barrier Dysfunction, *n* = 117				
	<30 nmol/L (*n* = 38)	30–50 nmol/L (*n* = 64)	>50 nmol/L (*n* = 190)	*p*-Value	<30 nmol/L (*n* = 19)	30–50 nmol/L (*n* = 36)	>50 nmol/L (*n* = 120)	*p*-Value	<30 nmol/L (*n* = 19)	30–50 nmol/L (*n* = 28)	>50 nmol/L (*n* = 70)	*p*-Value
Q_ALB_	7.3	7.2	6.3	<0.001	5.3	5.8	5.2	0.537	13.0	11.5	10.3	0.053
	(5.1–13.0)	(5.2–11.0) ^#^	(4.7–9.3) ^+^		(3.4–6.8)	(3.8–6.7)	(3.9–6.0)		(9.9–23.1)	(9.5–16.7)	(8.6–13.6)	
Q_25(OH)D_	5.3	4.1	3.1	<0.001	3.9	3.4	2.5	<0.001	9.3	5.6	5.0	0.004
	(3.6–10.5)	(3.1–5.8) ^#^	(2.3–4.8) ^+^		(2.3–5.3)	(2.4–4.1) ^#^	(2.0–3.4) ^+^		(5.5–13.4)	(4.5–8.9)	(4.0–6.7) ^+^	
Q_24,25(OH)2D3_	5.9	4.2	2.8	<0.001	4.2	3.5	2.4	<0.001	9.5	4.9	4.2	<0.001
	(3.9–9.5) *	(3.1–5.5) ^#^	(2.1–3.9) ^+^		(2.8–5.6)	(2.6–4.4) ^#^	(1.9–2.9) ^+^		(6.0–13.3) *	(4.1–7.4) ^#^	(3.4–5.6) ^+^	

CSF/serum albumin (Q_ALB_), 25(OH)D (Q_25(OH)D_) and 24,25(OH)_2_D_3_ (Q_24,25(OH)2D3_) according to tertiles of 25(OH)D serum levels. Data are given in medians and interquartile ranges (Q1–Q3). The Kruskal–Wallis test was used for subgroup comparisons. A *p*-value < 0.05 was considered statistically significant. Bonferroni correction for subgroups (*p* < 0.05): * <30 vs. 30–50 nmol/L; ^#^ 30–50 vs. >50 nmol/L; ^+^ <30 vs. >50 nmol/L. CSF, cerebrospinal fluid.

## Data Availability

The data are stored in a document file at our institution accessible only by the first author.

## Supplementary Materials

The following are available online at https://www.mdpi.com/article/10.3390/biom11091288/s1, Figure S1: Representative chromatograms of (A) 25(OH)D3 (2.6 nmol/L), (B) 25(OH)D2 (0.9 nmol/L) and (C) 24,25(OH)2D3 (0.07 nmol/L) (on the left side), in a native liquor sample and their deuterated internal standards (d6-25OH)D3, d3-25(OH)D2 and d6-24,25(OH)2D3 each with 150 nmol/L) (on the right side) obtained by LC-MS/MS in multiple reaction monitoring (MRM) mode.
