# Supplementary material for: A Novel Method for the Determination of Vitamin D Metabolites Assessed at the Blood-Cerebrospinal Fluid Barrier
Source: Biomolecules. 2021 Aug 29;11(9):1288. doi: 10.3390/biom11091288 (PMC8470512; doi:10.3390/biom11091288)
Supplement: Supplementary file 1 [file biomolecules-11-01288-s001.zip › biomolecules-1352957-supplementary.pdf]

Supplementary material

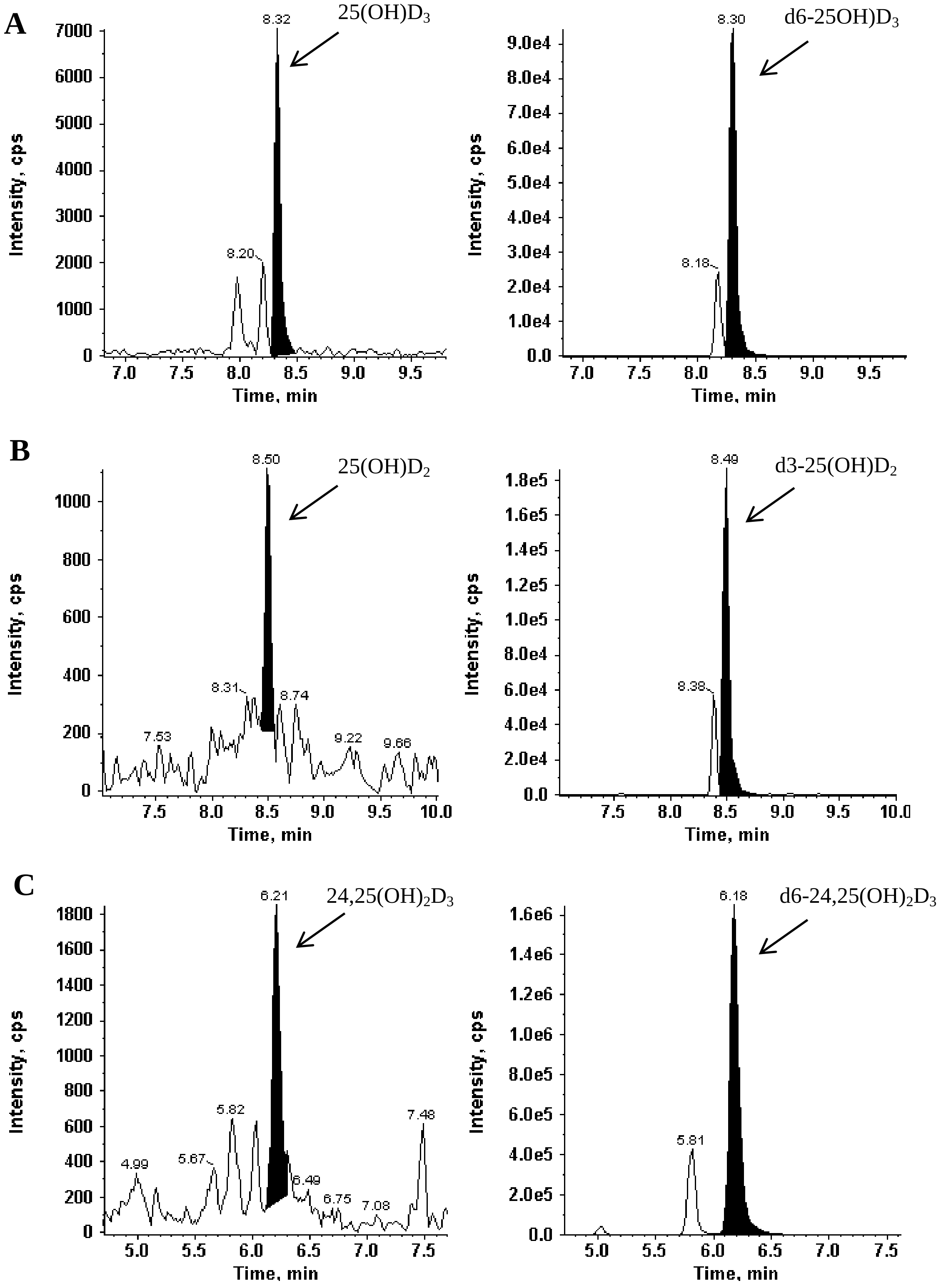

Figure S1. Representative chromatograms of (A) 25(OH)D_3 (2.6 nmol/L), (B) 25(OH)D_2 (0.9 nmol/L) and (C) 24,25$(OH)_2D_3$ (0.07 nmol/L) (on the left side), in a native liquor sample and their deuterated internal standards (d6-25OH)D_3, d3-25(OH)D_2 and d6-24,25$(OH)_2D_3$ each with 150 nmol/L) (on the right side) obtained by LC-MS/MS in multiple reaction monitoring (MRM) mode. The black coloured peak represents the target analyte in the chromatograms. cps, counts per second
